# QTL Mapping of Adult-Plant Resistance to Leaf Rust in the Wheat Cross Zhou 8425B/Chinese Spring Using High-Density SNP Markers

**DOI:** 10.3389/fpls.2017.00793

**Published:** 2017-05-16

**Authors:** Peipei Zhang, Guihong Yin, Yue Zhou, Aiyong Qi, Fengmei Gao, Xianchun Xia, Zhonghu He, Zaifeng Li, Daqun Liu

**Affiliations:** ^1^College of Plant Protection, Agricultural University of HebeiBaoding, China; ^2^Zhoukou Academy of Agricultural SciencesZhoukou, China; ^3^Department of Biochemistry, Baoding UniversityBaoding, China; ^4^Institute of Crop Science, National Wheat Improvement Center – Chinese Academy of Agricultural SciencesBeijing, China

**Keywords:** APR, *Puccinia triticina*, single nucleotide polymorphism, simple sequence repeat marker, *Triticum aestivum*

## Abstract

Wheat leaf rust is an important disease worldwide. Growing resistant cultivars is an effective means to control the disease. In the present study, 244 recombinant inbred lines from Zhou 8425B/Chinese Spring cross were phenotyped for leaf rust severities during the 2011–2012, 2012–2013, 2013–2014, and 2014–2015 cropping seasons at Baoding, Hebei province, and 2012–2013 and 2013–2014 cropping seasons in Zhoukou, Henan province. The population was genotyped using the high-density Illumina iSelect 90K SNP assay and SSR markers. Inclusive composite interval mapping identified eight QTL, designated as *QLr.hebau-2AL*, *QLr.hebau-2BS*, *QLr.hebau-3A*, *QLr.hebau-3BS*, *QLr.hebau-4AL*, *QLr.hebau-4B*, *QLr.hebau-5BL,* and *QLr.hebau-7DS*, respectively. *QLr.hebau-2BS*, *QLr.hebau-3A*, *QLr.hebau-3BS,* and *QLr.hebau-5BL* were derived from Zhou 8425B, whereas the other four were from Chinese Spring. Three stable QTL on chromosomes 2BS, 4B and 7DS explained 7.5–10.6%, 5.5–24.4%, and 11.2–20.9% of the phenotypic variance, respectively. *QLr.hebau-2BS* in Zhou 8425B might be the same as *LrZH22* in Zhoumai 22; *QLr.hebau-4B* might be the residual resistance of *Lr12*, and *QLr.hebau-7DS* is *Lr34*. *QLr.hebau-2AL*, *QLr.hebau-3BS*, *QLr.hebau-4AL,* and *QLr.hebau-5BL* are likely to be novel QTL for leaf rust. These QTL and their closely linked SNP and SSR markers can be used for fine mapping, candidate gene discovery, and marker-assisted selection in wheat breeding.

## Introduction

Wheat is among the most important food crops (Curtis and Halford, 2014). Leaf rust (LR), caused by *Puccinia triticina* (*Pt*), is an important disease on wheat. LR occurred severely in 1969, 1973, 1975, 1979, and 2012 in China and caused serious yield loss (Dong, 2001; Zhou et al., 2013). Although LR can be controlled by fungicides overuse may lead to fungicide residues in the grain, or resistance to fungicides followed by a resurgence of the disease under favorable conditions (Luo, 2009). Therefore, planting resistant cultivars is the most effective, economic and environmentally safe mean of controlling the disease.

There are two kinds of resistance, viz. race specific and race non-specific to wheat LR. Race specific resistance is often controlled by a single gene or simple gene combinations and elicits a HR. This kind of resistance is often not durable as it is readily overcome by new pathotypes that lack the corresponding avirulence factors. In contrast, race non-specific resistance, termed as APR, slow rusting or partial resistance, typically reduces growth and reproduction of the pathogen on adult plants. It is usually controlled by several minor genes, and tends to be more durable compared with race specific resistance.

To date, more than 100 LR genes have been documented, and 76 have been cataloged (McIntosh et al., 2016). Most of these genes are race specific and can be overcome easily by new pathogen pathotypes. Only four known slow rusting resistance genes, viz. *Lr34*, *Lr46*, *Lr67* and *Lr68*, have been cataloged at present (Dyck, 1977; Singh et al., 1998; Herrera-Foessel et al., 2011; Herrera-Foessel et al., 2012; Li et al., 2014). Besides the four cataloged slow rusting genes, 80 other APR QTL for LR have been mapped on 16 wheat chromosomes (Li et al., 2014). Although minor gene resistance tends to be more durable than major gene resistance, it also might be overcome by slow evolution in the pathogen populations (McDonald and Linde, 2002). Therefore, it is very important to identify more APR gene in wheat cultivars for controlling wheat rusts in China.

Molecular markers have been widely used in mapping and cloning resistance genes. SSR markers are widely used in linkage mapping due to advantages of co-dominance, stability, high polymorphism, chromosome specificity, and ease of manipulation (Röder et al., 1998). The SNP gene-chip technology developed quickly recently, and it provides a superior way for gene mapping due to its higher accuracy and density than other markers (Yu et al., 2011). A combined use of SSR and SNP markers is rational in that SSR might act as a framework to anchor SNP to the chromosomes. The map provides connections between SNP and SSR markers and can be used for comparative mapping of QTL linked to SSR markers reported before (Li et al., 2015).

The Chinese wheat line Zhou 8425B, developed by the Zhoukou Academy of Agricultural Sciences (ZAAS) in 1984, is still high resistant to wheat rusts and powdery mildew in the field at present. During the past 20 years, about 100 cultivars were derived from this line and had been planted over 33 million ha in China (Yin et al., 2009). In our previous work, a seedling major gene *LrZH84* was mapped on chromosome 1BL in Zhou 8425B (Zhao et al., 2008). The aim of present study was to identify APR QTL to LR and their closely linked molecular markers for MAS in wheat breeding.

## Materials and Methods

### Wheat Materials and *P. triticina* Pathotypes

A total of 244 F_8_ RILs derived from Zhou 8425B/Chinese Spring cross were used to map QTL for APR to LR. Zhou 8425B, Chinese Spring, Zhoumai 22, and 36 differential lines were included in the seedling tests with 14 Chinese *Pt* pathotypes (**Table 1**). The pathotypes were named following the *Pt* coding system described by Long and Kolmer (1989).

**Table 1:** Seedling infection types on Zhou 8425B, Chinese Spring, Zhoumai 22 and 36 wheat lines with known *Lr* genes when tested with 14 Chinese *Puccinia triticina* pathotypes.

Tester	*Lr* gene	Infection types to *Puccinia triticina* pathotypes													
		PHKS	MHJS^a^	FHDQ 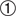	FGBQ	FHBQ	FHDQ 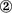	THJL^a^	FHDR	FGDQ	FHDS	THJP	TGTT	PHGP^a^	THJC
RL6003	*Lr1*	4	4	;	;	;	;	4	0	;	0	4	4	4	4
RL6016	*Lr2a*	;	;	1+	;	;	1	3	;	;	2	3	3	;	4
RL6078	*Lr26*	4	4	4	1	4	4	4	4	1	4	4	;1	4	4
RL6007	*Lr3ka*	X	X	;	;	;	;	1	;	;	;	1	4	;	X
RL6053	*Lr11*	4	4	1	;	;	1+	3+	1	1	2	4	3+	4	4
RL6008	*Lr17*	4	3+	3+	2	2	3+	4	3+	4	4	4	4	2+	4
RL6049	*Lr30*	3C	1	1	;	;	;	1	;	;	;	;	4	;	1
RL6004	*Lr10*	3	3	4	4	4	4	2	4	4	4	2+	4	1	X
RL6013	*Lr14a*	4	4	X	X	X	X	X	X	2	4	4	4	3+	X
RL6009	*Lr18*	1	1+	2	2	2	2	1+	4	2+	2	4	3+	3C	3
RL6019	*Lr2b*	1	0;	4	;	3	3+	4	3	3+	3+	2	4	3C	4
RL6052	*Lr15*	1	;	;	;	;	;	4	1	;	;	4	3+	4	4
RL6092	*Lr20*	4	4	;	;	;	;	;	;	;	;	4	1	4	;
RL6043	*Lr21*	4	2	2	;	2+	3	;	1	;	1+	;	3	1	1
E84018	*Lr36*	4	2	1+	;	2	2	1	2	2+	3	2+	3+	2+	3+
RL6147	*Lr44*	1	;	4	4	4	4	1	4	4	4	;	1+	;1	1
RL6144	*Lr45*	4	4	4	4	4	4	;	4	4	4	4	;	;	;
Zhou 8425B		4	4	2	;	2	4	4	2+	;	2	2+	2	4	;
Chinese Spring		4	4	4	4	4	3+	4	4	4	4	4	3+	4	4
Zhoumai 22	*Lr26*+ *LrZH22*	4	3	1+	;1	1	4	4	2	;	;	3C	1	3	0
Zhengzhou 5389		4	4	4	4	4	4	4	4	4	4	4	4	4	4

^a^*Pathotypes were used in field trials*.

### Evaluation of Seedling Responses in the Greenhouse

Seedlings were planted in a growth chamber (30 cm × 50 cm). Inoculation of seedlings was carried out by the method provided by Li et al. (2010). ITs were recorded 10–14 days after inoculation based on the 0–4 Stakman scale modified by Roelfs et al. (1992). The gene postulation was conducted using the method reported by Dubin et al. (1989). The Zhou 8425B/Chinese Spring RIL population was also inoculated with the pathotype FHDQ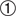 to verify the seedling gene in Zhou 8425B.

### Leaf Rust Tests in the Field

Zhou 8425B, Chinese Spring and the 244 F_8_ RILs from the cross Zhou 8425B/Chinese Spring were evaluated for APR for LR in the field at Baoding, Hebei province during the 2011–2012, 2012–2013, 2013–2014, and 2014–2015 cropping seasons and Zhoukou, Henan province during the 2012–2013 and 2013–2014 cropping seasons. The field test designed followed Qi et al. (2016). Spreader rows of susceptible line Zhengzhou 5389 were sown perpendicular and adjacent to the test lines to aid the spread of spores. Equal amounts of *Pt* pathotypes PHGP, MHJS, and THJL (virulent on seedlings of Zhou 8425B and Chinese Spring) were used for field inoculation. Field inoculation was conducted by the method provided by Zhou et al. (2014). Four weeks after inoculation LR severities were recorded three times at weekly intervals using the modified Cobb scale (Peterson et al., 1948). MDS at Baoding in Hebei in the 2011–2012, 2012–2013, 2013–2014, and 2014–2015 cropping seasons and Zhoukou in Henan in the 2012–2013 and 2013–2014 cropping seasons will hereafter be referred to as 2012BD, 2013BD, 2014BD, 2015BD, 2013ZK, and 2014ZK, respectively; these were used for statistical and QTL analysis.

### Statistical Analysis

Analyses of variance (ANOVA) were conducted by IBM SPSS Statistics 19.0 software. The correlation coefficients of phenotypic data between MDS in different environments were calculated by the Microsoft Excel analytical tool. Broad-sense heritabilities (*h^2^*) for LR resistance were calculated by the formula: *h^2^* = *σ_g_^2^*/(*σ_g_^2^*+*σ_ge_^2^*/*e*+*σ*_ε_*^2^*/*re*) (Zhou et al., 2014).

### Genotyping Using SNP and SSR Markers

Genomic DNA was extracted from 10 non-infected seedling leaves of each line including the parents using the CTAB method (Sharp et al., 1988). Zhou 8425B, Chinese Spring and the 244 RILs were genotyped by the 90K iSelect SNP array in our previous study (Gao et al., 2015). In addition, 26 SSR markers were also used to genotype the whole population for further linkage and QTL mapping in the present study.

### QTL Analysis

Linkage maps were constructed using SNP markers combined with SSRs. The software QTL IciMapping 3.1 was used to detect APR QTL (Li et al., 2007). Phenotypic values of all lines in each environment were used for QTL detection. The procedures for QTL detection and digenic interactions analysis between non-allelic QTL were similar with Zhou et al. (2014) and Qi et al. (2016).

## Results

### Resistance Genes Postulated from Seedling Reactions

The different ITs of 36 wheat differential lines with known *Lr* genes provided an ability to postulate 17 LR genes, viz. *Lr1*, *Lr2a*, *Lr26*, *Lr3ka*, *Lr11*, *Lr17*, *Lr30*, *Lr10*, *Lr14a*, *Lr18*, *Lr2b*, *Lr15*, *Lr20*, *Lr21*, *Lr36*, *Lr44*, and *Lr45*, when inoculated with 14 *Pt* pathotype (**Table 1**). Ten genes, viz. *Lr9*, *Lr19*, *Lr24*, *Lr28*, *Lr29*, *Lr39*, *Lr42*, *Lr47*, *Lr51*, and *Lr53*, showed resistance to all the tested pathotypes. It is not possible to postulate genes *Lr2c*, *Lr3a*, *Lr3b*, *Lr13*, *Lr14b*, *Lr16*, *Lr23*, *Lr33*, and *LrB* because these genes were susceptible to most pathotypes. Zhou 8425B showed a similar response pattern to Zhoumai 22 which carries *LrZH22* (Wang et al., 2016). Zhou 8425B is one of the parents of Zhoumai 22, so *LrZH22* in Zhoumai 22 was likely derived from Zhou 8425B. In previous report Zhou 8425B also contained *Lr26* and *LrZH84* (Zhao et al., 2008). In the seedling test Zhou 8425B was resistant to three *Pt* pathotypes avirulent to *Lr26*, which further confirmed that Zhou 8425B contained *Lr26*.

### Phenotypic Evaluation for Leaf Rust in the Field

Leaf rust developed well in all environments. As pathotypes used in the field were virulent on seedlings of both parents, low disease severities presumably resulted from slow rusting resistance genes. The MDS of Zhengzhou 5389 ranged from 70 to 100% across environments. Zhou 8425B had mean MDS scores of 10% across six environments, whereas Chinese Spring showed a MDS of 15%. MDS in the RILs ranged from 1 to 100% across all environments exhibiting significant differences among genotypes. The frequency distribution of LR MDS in each environment showed a continuous distribution skewed toward resistance (Supplementary Figure S1), indicating polygenic inheritance. Correlation coefficients for the population ranged from 0.59 to 0.82 across different environments (*P* < 0.0001) (**Table 2**). Mean MDS across all environments was 16.7%. Broad-sense heritability of MDS across six environments was 0.77. ANOVA confirmed significant variation among the RILs (**Table 3**).

**Table 2:** Pearson correlation coefficients (r) for two-way comparisons of leaf rust (LR) severity data from different environments.

	2012BD	2013BD	2014BD	2015BD	2013ZK	2014ZK
2012BD		0.75^∗∗^	0.77^∗∗^	0.66^∗∗^	0.72^∗∗^	0.61^∗∗^
2013BD			0.82^∗∗^	0.59^∗∗^	0.74^∗∗^	0.74^∗∗^
2014BD				0.65^∗∗^	0.76^∗∗^	0.74^∗∗^
2015BD					0.60^∗∗^	0.59^∗∗^
2013ZK						0.69^∗∗^
2014ZK						

*^∗∗^Significant at *P* = 0.01*.

**Table 3:** Analysis of variance of maximum disease severities (MDS) for leaf rust in the population of Zhou 8425B/Chinese Spring.

Source of variation	*df*	Sum of squares	Mean square	*F*-value	*P*
Genotype	243	1344088.52	5531.23	14.40^∗∗^	<0.0001
Environment	5	38768.75	7753.75	20.27^∗∗^	<0.0001
Replicates	1	186.77	186.77	0.49	0.48
Genotype × Environment	1215	1562882.65	1286.32	3.36^∗∗^	<0.0001
Error	1463	559711.61	382.58		

*^∗∗^Significant at *P* = 0.01*.

### Linkage Map Construction

**FIGURE 1:**
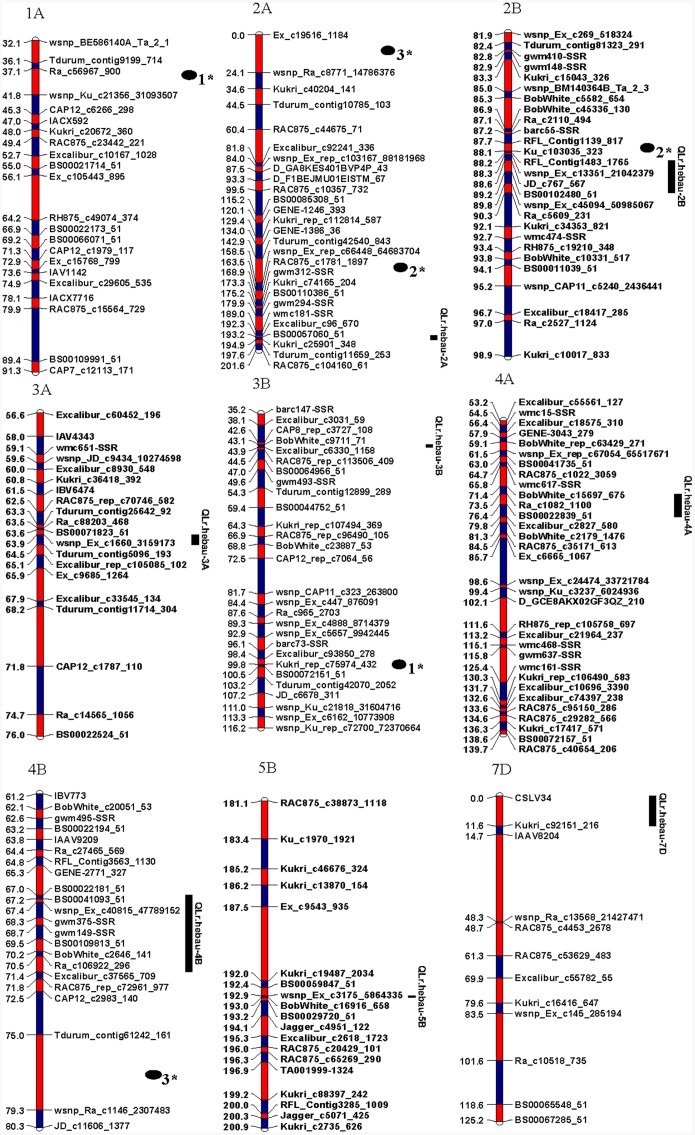
**Simplified genetic maps of nine chromosomes showing QTL for leaf rust (LR) resistance in the Zhou 8425B/Chinese Spring population**. The bar length of each chromosome do not represent the same genetic distance. The epistasis QTLs also list on the figure. 1^∗^, 2^∗^, and 3^∗^ indicate interactions in 2012BD, 2014BD, and 2015BD, respectively, on the map.

A total of 21 linkage groups corresponding to the 21 hexaploid wheat chromosomes were constructed from 5,636 high-quality polymorphic SNP markers (Gao et al., 2015) and 26 SSR markers (**Figure 1**).

### Resistance of *LrZH84* in the Field

Zhou 8425B was known to carry *Lr26* and *LrZH84* (Zhao et al., 2008). *Lr26* have lost resistance to most of *Pt* pathotypes in China. *LrZH84* was mapped on 1BL, and linked to SSR markers *gwm582* and *barc8* with genetic distances of 3.9 and 5.2 cM, respectively (Zhao et al., 2008). In the present study, two SSR markers *gwm582* and *barc8* linked to *LrZH84* were used to test the entire RIL population to identify the effect of *LrZH84* at adult-plant stage; the result showed that lines with *LrZH84* had lost resistance to the mixed virulent pathotypes in the field (data not shown). The APR in Zhou 8425B was conferred by other LR resistance genes.

### QTL for LR Resistance

Eight putative LR APR QTL were identified on chromosomes 2AL, 2BS, 3A, 3BS, 4AL, 4B, 5BL, and 7DS (**Table 4** and **Figure 1**) based on the MDS data, and were designated as *QLr.hebau-2AL*, *QLr.hebau-2BS*, *QLr.hebau-3A*, *QLr.hebau-3BS*, *QLr.hebau-4AL*, *QLr.hebau-4B*, *QLr.hebau-5BL,* and *QLr.hebau-7DS*, respectively. The QTL on 2BS, 3A, 3BS, and 5BL were derived from Zhou 8425B, whereas those on 2AL, 4AL, 4B, and 7DS were from Chinese Spring. As expected there was no evidence of any contributions from *Lr26* or *LrZH84*.

**Table 4:** Quantitative trait locus/loci for MDS to leaf rust by ICIM in the RIL population from Zhou 8425B/Chinese Spring.

Environment	QTL^a^	Position	Marker interval	LOD^b^	PVE (%)^c^	Add^d^
^∗^2012BD	*QLr.hebau-2BS*	89	JD_c767_567 – BS00102480_51	5.2	7.5	-4.9
	*QLr.hebau-3BS*	43	BobWhite_c9711_71 – Excalibur_c6330_1158	2.8	3.5	-3.3
	*QLr.hebau-7DS*	6	Kukri_c92151_216 – CSLV34	9.9	17.8	7.5
^∗^2013BD	*QLr.hebau-2AL*	193	Excalibur_c96_670 – BS00057060_51	3.0	4.8	6.1
	*QLr.hebau-2BS*	90	wsnp_Ex_c45094_50985067 – Ra_c5609_231	5.6	8.0	-8.1
	*QLr.hebau-4B*	69	*gwm149* – BS00109813_51	3.9	5.5	6.7
	*QLr.hebau-7DS*	7	Kukri_c92151_216 – CSLV34	10.8	17.0	11.6
^∗^2013ZK	*QLr.hebau-2BS*	89	JD_c767_567 – BS00102480_51	7.8	10.6	-5.8
	*QLr.hebau-4B*	71	Ra_c106922_296 – Excalibur_c37565_709	6.0	7.9	5.0
	*QLr.hebau-5BL*	193	wsnp_Ex_c3175_5864335 – BobWhite_c16916_658	4.4	5.4	-4.1
	*QLr.hebau-7DS*	9	Kukri_c92151_216 – CSLV34	8.9	12.4	6.3
^∗^2014BD	*QLr.hebau-2BS*	89	JD_c767_567 – BS00102480_51	8.3	10.0	-7.7
	*QLr.hebau-3A*	64	wsnp_Ex_c1660_3159173 – Tdurum_contig5096_193	3.0	2.9	-4.1
	*QLr.hebau-4AL*	73	BobWhite_c15697_675 – Ra_c1082_1100	3.4	3.4	4.4
	*QLr.hebau-4B*	69	*gwm149* – BS00109813_51	8.4	8.8	7.1
	*QLr.hebau-7DS*	8	Kukri_c92151_216 – CSLV34	11.0	12.7	8.7
^∗^2014ZK	*QLr.hebau-2BS*	89	JD_c767_567 – BS00102480_51	5.9	9.4	-7.2
	*QLr.hebau-4AL*	78	BS00022839_51 – Excalibur_c2827_580	2.9	7.5	6.6
	*QLr.hebau-4B*	68	wsnp_Ex_c40815_47789152 – *wms375*	5.9	9.0	6.9
	*QLr.hebau-7DS*	8	Kukri_c92151_216 – CSLV34	6.6	11.2	7.9
^∗^2015BD	*QLr.hebau-2AL*	191	*wmc181* – Excalibur_c96_670	2.5	4.5	5.5
	*QLr.hebau-4B*	67	BS00022181_51 – BS00041093_51	17.1	24.4	12.4
	*QLr.hebau-7DS*	9	Kukri_c92151_216 – CSLV34	12.4	20.9	11.6
^∗^Average MDS	*QLr.hebau-2AL*	193	Excalibur_c96_670 – BS00057060_51	5.2	6.6	5.0
	*QLr.hebau-2BS*	89	JD_c767_567 – BS00102480_51	6.0	7.5	-5.4
	*QLr.hebau-4AL*	78	BS00022839_51 – Excalibur_c2827_580	3.1	5.9	5.0
	*QLr.hebau-4B*	69	*gwm149* – BS00109813_51	7.7	8.6	5.7
	*QLr.hebau-7DS*	8	Kukri_c92151_216 – CSLV34	14.4	18.6	8.6

^a^*QTL that overlap in the one-log support confidence intervals were assigned the same symbol. ^b^Logarithm of odds (LOD) score. ^c^Percentages of phenotypic variance explained by individual QTL. ^d^Additive effect of resistance allele*.

*QLr.hebau-7DS*, in the marker interval Kukri_c92151_216 – csLV34 was stably detected in all environments, explaining 17.8, 17.0, 12.4, 12.7, 11.2, 20.9, and 18.6% of the phenotypic variances in 2012BD, 2013BD, 2013ZK, 2014BD, 2014ZK 2015BD and average MDS, with additive effects from Chinese Spring of 7.5, 11.6, 6.3, 8.7, 7.9, 11.6, and 8.6, respectively.

The second QTL *QLr.hebau-2BS* identified in 2012BD, 2013BD, 2013ZK, 2014BD, 2014ZK and average MDS accounted for 7.5, 8.0, 10.6, 10.0, 9.4, and 7.5% of the phenotypic variances, respectively. The additive effects from Zhou 8425B were 4.9, 8.1, 5.8, 7.7, 7.2, and 5.4, respectively. To further confirm the relationship between *LrZH22* and *QLr.hebau-2BS*, the pathotype FHDQ avirulent to *LrZH22* was used to inoculate the whole population at the seedling stage. The RIL population segregated 112 resistant lines with IT 1–2, 126 susceptible lines with IT 3–4 and 6 segregated lines, indicating single gene *LrZH22* conferred seedling resistance to FHDQ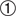 (χ^2^ = 2.09, 2 df, *P* > 0.25). Then the phenotype data was combined with genotype data for mapping *LrZH22* using software Joinmap 4.0. The result showed that the two closest flanking SNP loci were JD_c767_567 and RFL_Contig1483_1765 with genetic distances of 0.3 and 0.6 cM, respectively; this places the *LrZH22* locus in the same vicinity as *QLr.hebau-2BS*. Thus *QLr.hebau-2BS* should be the same gene in Zhoumai 22. The APR gene effect on 2BS is likely to be residual resistance from *LrZH22* (Wang et al., 2016).

*QLr.hebau-4B* from Chinese Spring in the marker interval BS00022181_51 – Excalibur_c37565_709 identified in 2013BD, 2013ZK, 2014BD, 2014ZK, 2015BD and average MDS explained 5.5, 7.9, 8.8, 9.0, 24.4, and 8.6% of the phenotypic variances, respectively. The additive effects were 6.7, 5.0, 7.1, 6.9, 12.4, and 5.7, respectively.

Two QTL were identified only in two environments and average MDS. *QLr.hebau-2AL* in the marker interval *wmc181* – BS00057060_51 explained 4.8, 4.5, and 6.6% of the phenotypic variance in 2013BD, 2015BD and average MDS with additive effects from Chinese Spring of 6.1, 5.5, and 5.0, respectively. Another QTL *QLr.hebau-4AL*, located in the region of BobWhite_c15697_675 – Excalibur_c2827_580, explained 3.4, 7.5, and 5.9% of the phenotypic variance in 2014BD, 2014ZK and average MDS, with additive effects from Chinese Spring of 4.4, 6.6, and 5.0, respectively. The effects of the two QTL need to verify using further evidence.

The three tentative QTL, viz. *QLr.hebau-3A*, *QLr.hebau-3BS* and *QLr.hebau-5BL*, were detected only in one environment, and their effects need to be further confirmed. *QLr.hebau-3A*, in marker interval wsnp_Ex_c1660_3159173 – Tdurum_contig5096_193 explained 2.9% of the phenotypic variance in 2014BD, with an additive effect from Zhou 8425B of 4.1. *QLr.hebau-3BS*, flanked by SNP markers BobWhite_c9711_71 and Excalibur_c6330_1158, explained 3.5% of the phenotypic variance in 2012BD, with an additive effect from Zhou 8425B of -3.3. *QLr.hebau-5BL*, in interval wsnp_Ex_c3175_5864335 – BobWhite_c16916_658, accounted for 5.5% of the phenotypic variance in 2013ZK, and the additive effect from Zhou 8425B was 4.1.

The total phenotypic variances explained by all QTL in a simultaneous fit ranged from 28.8 to 49.8% across environments, indicating significant effects of the QTL in reducing LR severity.

### Epistasis of QTL for Leaf Rust Resistance

Three epistatic QTL were identified for the LR resistance. The variation explained ranged from 7.5 to 18.4% (**Table 5** and **Figure 1**). The first interaction was detected between the chromosome 1AS and 3B in 2012BD, explaining 7.5% of the phenotypic variance with the additive effect of -5.1. The second interaction was detected between chromosome 2AL and 2BS in 2014BD, explaining the phenotypic variance of 15.1% with the additive effect of -9.5. The QTL on 2BS located at the similar position as *QLr.hebau-2BS*. The last interaction is detected between chromosome 2AS and 4B in 2015BD, which explained 18.4% of the phenotypic variance with the additive effect of 9.7.

**Table 5:** Epistastic QTL for leaf rust resistance in the Zhou 8425B/Chinese Spring population.

Env^a^	Chr^b^	Po^c^	Marker interval	Chr	Po	Marker interval	LOD	PVE (%)	AA^d^
2012BD	1AS	40	Ra_c56967_900 – wsnp_Ku_c21356_31093507	3B	100	Kukri_rep_c75974_432 – BS00072151_51	5.3	7.5	-5.1
2014BD	2AL	155	Tdurum_contig42540_843 – wsnp_Ex_rep_c66448_64683704	2BS	88	RFL_Contig1139_817 – Ku_c103035_323	6.1	15.1	-9.5
2015BD	2AS	10	Ex_c19516_1184 – wsnp_Ra_c8771_14786376	4B	75	Tdurum_contig61242_161 – wsnp_Ra_c1146_2307483	5.7	18.4	9.7

^a^*Environment; ^b^Chromosome on which the QTL was located; ^c^Position on the chromosome; ^d^AA is the effect of additive × additive interaction between two intervals, the positive AA value indicates that effect of the parent-type is greater than that of the recombinant-type, and the negative AA value indicates that effect of the parent-type is less than that of the recombinant-type*.

## Discussion

### The Major QTL

In the present research five major QTL were identified by QTL analysis using the average MDS from different environments.*QLr.hebau-2AL.*

*QLr.hebau-2AL* was mapped in the marker interval *wmc181* – BS00057060_51. Three known LR QTL, viz. *QLr.cimmyt-2AL* (Rosewarne et al., 2012), *QLr.sfr-2AL* (Schnurbusch et al., 2004) and *QLr.ubo-2A* (Maccaferri et al., 2008), were located on chromosome 2AL. *QLr.cimmyt-2AL* and *QLr.sfr-2AL* were mapped at 63 cM on 2A, and *QLr.ubo-2A* was at the end of 2AL (143 cM) (Li et al., 2014). *QLr.hebau-2AL* linked to *wmc181* was mapped at 103 cM on 2A based on the consensus map of Somers et al. (2004). Therefore, *QLr.hebau-2AL* appears to be a new QTL.

#### QLr.hebau-2BS

Six known *Lr* genes, viz. *Lr13*, *Lr16*, *Lr23*, *Lr35*, *Lr48,* and *LrZH22* (Li et al., 2014; Wang et al., 2016) are located on chromosome 2BS. *QLr.hebau-2BS* was closely linked with SSR markers *barc55* and *wmc474*, in the same position as *LrZH22* (Wang et al., 2016). In the seedling tests Zhou 8425B was postulated to contain *LrZH22*, a temperature-sensitive gene on chromosome 2BS in Zhoumai 22 (Wang et al., 2016), and *QLr.hebau-2BS* is likely to be residual resistance from *LrZH22*.

#### QLr.hebau-4AL

*QLr.hebau-4AL* from Chinese Spring was located between BobWhite_c15697_675 and Excalibur_c2827_580, and also closely linked to SSR marker *wmc617*. No LR resistance gene was previously reported in this position (Li et al., 2014), hence *QLr.hebau-4AL* is a new APR QTL.

#### QLr.hebau-4B

Four known LR resistance genes, viz. *Lr12* (Singh and Bowden, 2011), *Lr25* (Singh et al., 2012), *Lr31*, and *Lr49* (Bansal et al., 2008) and three APR QTL viz. *QLr.sfrs-4B* (Messmer et al., 2000), *QLr.pbi-4BL* (Singh et al., 2009), and *QLr.cimmyt-4BL* (William et al., 2006) have been mapped on chromosome 4B. In the present study *QLr.hebau-4B* was detected in Chinese Spring, which carries the race-specific APR gene *Lr12*. *QLr.hebau-4B* was flanked by SSR loci *Xgwm149* and *Xgwm375*, which were closely linked to *Lr12* with genetic distances of 1.9 and 3.1 cM, respectively. In the field test Chinese Spring was susceptible to inoculated mixture of pathotypes with IT 4, indicating that *Lr12* in Chinese Spring has lost resistance to the mixed pathotypes, and the minor effect of *QLr.hebau-4B* is likely to be the residual effect from the race-specific gene *Lr12*. However, in another field trial the MDS of RL6011 (*Lr12*) was 5% with IT 2 and Thatcher (*Lr22*) was 70% with IT 4 (data not shown); the relationship between *QLr.hebau-4B* and *Lr12* in RL6011 is unclear and needs to be tested in the future.

#### QLr.hebau-7DS

The known pleiotropic APR gene *Lr34*/*Yr18*/*Pm38*/*Sr57* was mapped on chromosome 7DS (Lillemo et al., 2008). In the present study, *QLr.hebau-7DS* detected from Chinese Spring was on chromosome 7DS, flanked by markers csLV34 (closely linked to *Lr34*) and Kukri_c92151_216. Chinese Spring contains *Lr34* (Piech and Supryn, 1978; Krattinger et al., 2009); *QLr.hebau-7DS* should be *Lr34*.

### The Tentative QTL

Three QTL can only be detected in few environment(s) and can’t be detected in Average MDS.

#### QLr.hebau-3A

Three QTL *QLr.ubo-3A*, *QLr.sfrs-3AL,* and *QLr.fcu-3AL* were located on chromosome 3A (Li et al., 2014). *QLr.hebau-3A* in the marker interval wsnp_Ex_c1660_3159173 – Tdurum_contig5096_193 was linked to SSR markers *wmc651* and *wmc264* with genetic distances of 4.6 and 13.6 cM, respectively. Based on the Somers map *QLr.hebau-3A* was located at a similar position to *QLr.ubo-3A* and *QLr.sfrs-3AL* but with different genetic background. The relationships among the three genes were unclear and need to be verified in future work.

#### QLr.hebau-3BS

*QLr.hebau-3BS* from Zhou 8425B was located in marker interval BobWhite_c9711_71 – Excalibur_c6330_1158; it was linked to SSR markers *barc147* and *gwm493*, at genetic distances of 7.4 and 6.9 cM, respectively, at the position 10 cM on the Somers map. Two QTL_,_ *QLr.sfrs-3B* (Messmer et al., 2000) and *QLr.fcu-3BL* (Chu et al., 2009), have been located on chromosome 3B. *QLr.fcu-3BL* was mapped on chromosome 3BL and *QLr.sfrs-3B* was mapped at the position 61 cM on 3B, which is near the centromere based on the Somers map. Therefore, *QLr.hebau-3BS* is likely to be new.

#### QLr.hebau-5BL

Three known LR QTL *QLr.hbau-5BL* (Zhang et al., 2009), *QLr.sfrs-5BL* (Messmer et al., 2000), and *QLr.fcu-5BL* (Chu et al., 2009) were located at a similar position of 140 cM on chromosome 5B (Li et al., 2014), about was 34.1 cM from *QLr.hebau-5BL* in the present study. Therefore *QLr.hebau-5BL* is likely to be new.

### QTL and Their Phenotypic Effects

The total phenotypic variances explained by all additive QTL ranged from 28.8 to 49.8% across environments. The effect of APR QTL can be easily influenced by environment (Zhang et al., 2009). For example, *QLr.hebau-4B* explained 5.5–24.4% of the phenotypic variance across different environments, indicating the QTL was not very stable among different environments. Although the phenotypic variance explained by the epistatic QTL was changed across different environments, the interaction between QTL also played an important role in the genetic control of quantitative traits (Roncallo et al., 2012). In this study, different epistatic QTL were detected in 2012BD, 2014BD and 2015BD, and explained 7.5–18.4% of the phenotypic variance. Some interactions didn’t involve the detected QTL, but their effects are just as important as some of the individual QTL in terms of LOD, PVE, and AA effect. These suggested that the epistasis as a genetic factor played an important role in the LR resistance. The additive and epistatic effects of QTL together contributed to the LR resistance.

## Conclusion

In this study, 8 QTL were detected in the Zhou 8425B/Chinese Spring cross. Among these, *QLr.hebau-2BS* might be the same as *LrZH22* in Zhoumai 22 (Wang et al., 2016). *QLr.hebau-4B* is likely to be the residual resistance of *Lr12*. *QLr.hebau-7DS* is *Lr34*. *QLr.hebau-2AL*, *QLr.hebau-3BS*, *QLr.hebau-4AL,* and *QLr.hebau-5BL* might be new APR QTL. The genes and closely linked markers can permit accurate selection in marker-assisted wheat breeding for durable resistance to LR.

## Ethics Statement

We declare that these experiments comply with the ethical standards in China.

## Author Contributions

PZ prepared phenotypic data in the field and drafted the initial manuscript. GY, YZ, and AQ prepared some phenotypic data and prepared some tables and figures. FG prepared genotypic data. XX and ZH played a major role in planning this study and drafted some sections of the manuscript. ZL and DL led the design and coordination of this study.

## Conflict of Interest Statement

The authors declare that the research was conducted in the absence of any commercial or financial relationships that could be construed as a potential conflict of interest.

## Abbreviations

APR: adult plant resistance
HR: hypersensitive response
IT: infection type
LOD: logarithm of odds
LR: leaf rust
MAS: marker-assisted selection
MDS: maximum disease severity
*Pt*: *Puccinia triticina*
PVE: phenotypic variance explained
QTL: quantitative trait locus/loci
RIL: recombinant inbred line
SNP: single nucleotide polymorphism
SSR: simple sequence repeat.
