# Supplementary material for: QTL Mapping of Adult-Plant Resistance to Leaf Rust in the Wheat Cross Zhou 8425B/Chinese Spring Using High-Density SNP Markers
Source: Front Plant Sci. 2017 May 16;8:793. doi: 10.3389/fpls.2017.00793 (PMC5432574; doi:10.3389/fpls.2017.00793)
Supplement: Supplementary file 1 [file Image_1.PDF]

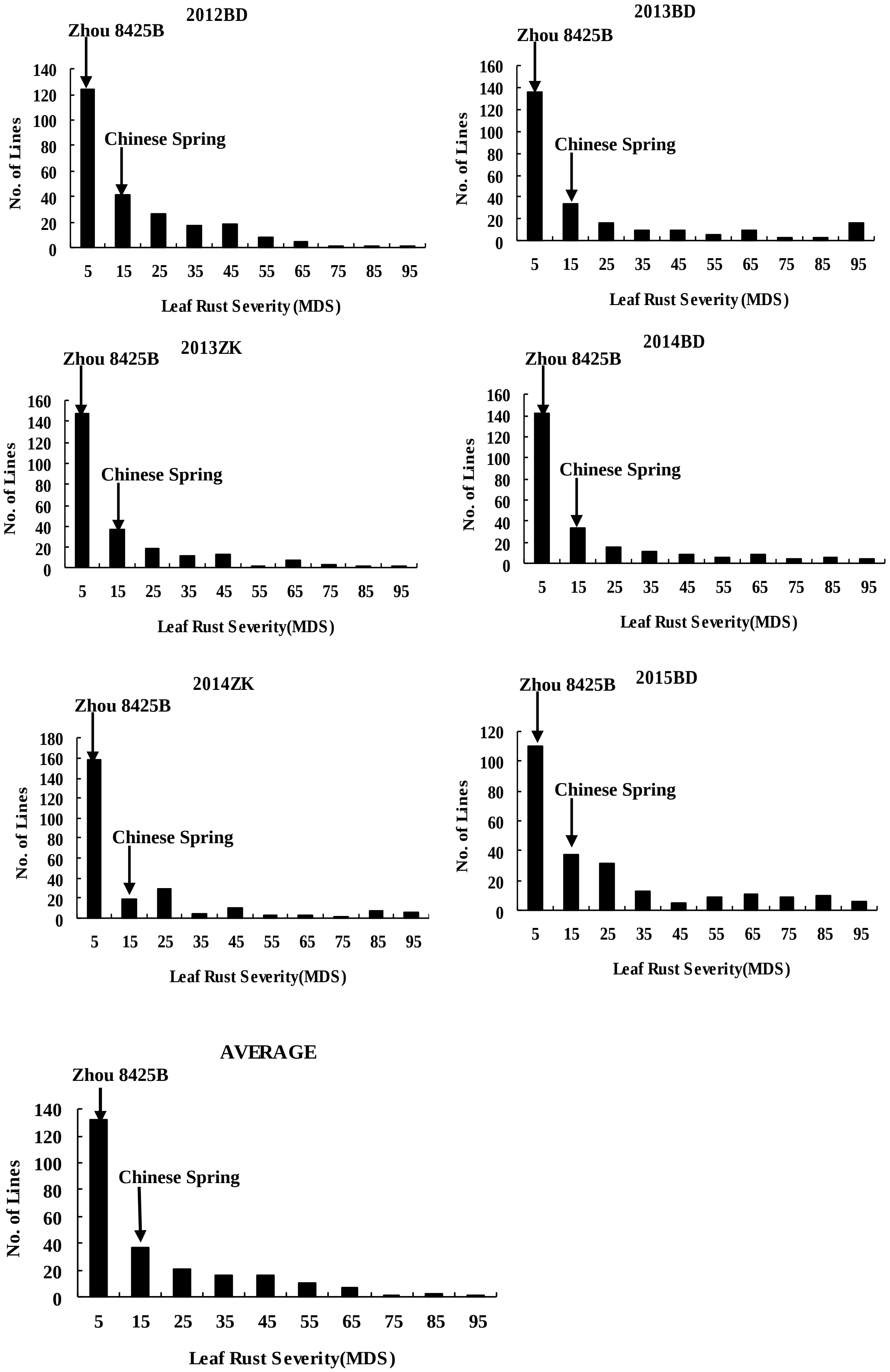
2012BD
2013BD
2013ZK
2014BD
2014ZK
2015BD
AVERAGE
Zhou 8425B
Chinese Spring
No. of Lines
Leaf Rust Severity (MDS)
5 15 25 35 45 55 65 75 85 95

Fig. S1 Frequency distributions of leaf rust MDS for the Zhou 8425B/Chinese Spring RIL population grown in Baoding (Hebei) and Zhoukou (Henan)
